# Measuring the impact of the French version of The Whiplash Book on both treatment approach and fear-avoidance beliefs among emergency physicians. A cluster randomized controlled trial

**DOI:** 10.1371/journal.pone.0229849

**Published:** 2020-03-18

**Authors:** Charlotte Lanhers, Stéphane Poizat, Bruno Pereira, Candy Auclair, Christophe Perrier, Jeannot Schmidt, Laurent Gerbaud, Emmanuel Coudeyre

**Affiliations:** 1 Department of Physical Medicine and Rehabilitation, Clermont-Ferrand University Hospital, Clermont-Ferrand, France; 2 Clermont-Ferrand Auvergne University, Clermont-Ferrand, France; 3 Innovation and Clinical Research, Clermont-Ferrand University Hospital, Clermont-Ferrand, France; 4 Department of Public Health, Biostatistics Unit, Clermont-Ferrand University Hospital, Clermont-Ferrand, France; 5 Emergency Department, Clermont-Ferrand University Hospital, Clermont-Ferrand, France; 6 INRA, Unity of Human Nutrition (UNH, UMR 1019), CRNH Auvergne, Clermont-Ferrand, France; University of Manitoba Faculty of Health Sciences, CANADA

## Abstract

**Background:**

Whiplash-associated disorders have been the subject of much attention in the scientific literature and remain a major public health problem.

**Objective:**

Measure the impact of a validated information booklet on the fear-avoidance beliefs of emergency physicians and their approach to management regarding the treatment of whiplash-associated disorders.

**Methods:**

A prospective cluster randomized controlled study conducted with a sample of emergency medicine physicians. Fear-avoidance beliefs were measured using The Whiplash Belief Questionnaire (WBQ) and Fear-Avoidance Beliefs Questionnaire (FABQ). We assessed the approach to management based on the prescription of pharmacological and non-pharmacological treatments based on the advice given to patients. The validated information booklet was the French version of The Whiplash Book. A set of questionnaires was sent to participants pre- and post-intervention. The experimental intervention was the provision of The Whiplash Book. The control arm did not receive any training or information.

**Results:**

Mean fears and beliefs scores on inclusion were high: WBQ = 19.09 (± 4.06); physical activity FABQ = 11.45 (± 4.73); work FABQ = 13.85 (± 6.70). Improvement in fear-avoidance beliefs scores being greater in the intervention group was further confirmed by the variation in WBQ (-20 [-32; -6] *vs*. -6 [-16; 9]; p = 0.06), physical activity FABQ (-70 [-86; -50] *vs*. -15 [-40; 11]; p < 0.001), and work FABQ (-40 [-71; 0] *vs*. 0 [-31; 50]; p = 0.02). The emergency physicians' initial approach to management was not consistent with current guidelines. Reading the French version of The Whiplash Book could contribute to changing their approach to management in several areas on intra-group analysis.

**Conclusion:**

The French version of The Whiplash Book positively influenced fear-avoidance beliefs among emergency physicians.

## Introduction

### Background

Whiplash is defined as minor indirect trauma to the cervical spine following a collision from behind at low speed or when stopped. It is an acceleration-deceleration mechanism of energy transfer to the neck that occurs while driving in a town or city [1]. The main obstacle to managing whiplash is that it is first and foremost associated with disorders and not a unique pathologic entity. This transfer of energy leads to bone or soft tissue-associated disorders that can result in a variety of clinical manifestations.

Whiplash trauma and the resulting whiplash-associated disorders have been the subject of much attention in the scientific literature and remain a major public health problem [2,3,4]. The rate of whiplash trauma occurrence is not well-known but seems to be about 1.33/1000 drivers per year in Australia [2]. Its incidence is said to be higher in women than men [2,3].

Most research teams posit multifactorial pathophysiology like that observed in other chronic pain conditions with no clearly defined nociceptive or neuropathic component present [5]. The most commonly encountered symptoms are neck pain, headache, low back pain, shoulder pain, as well as visual impairment [6]. Patients can be categorized by symptom grade according to the Quebec Task Force on Whiplash-Associated Disorders classification [7]. After a whiplash trauma, recovery rates across the cohorts were highly variable; likely this inconsistency is due to the number and variety of measures used to measure recovery. Between 38% and 55% of patients are asymptomatic at 1 month after the trauma, 65% at 12 months, and 75% at 5 years [8,9]. However, it is not unusual for neck pain to become chronic, and when this occurs, there may be serious consequences on a social, professional and financial level [9].

Of the demographic and accident logical factors most often associated with chronic neck pain are the following parameters: initial pain intensity, a high number of initial symptoms, anxiety, and severity of the injury as perceived by the patient [8,10]. Adverse prognostic factors vary, but the consensus in systematic reviews is high initial pain and disability and post-traumatic stress [9,11,12,13]. Even though the role of the initial episode should not be ignored, progression to chronic pain is probably multifactorial, and, like non-specific low back pain, psycho-social factors, and in particular patient fear-avoidance beliefs [14,15], seem to play an relevant role, as do environmental factors. The professional consensus is that it is useful to provide targeted information at an early stage in whiplash settings in order to reduce unhelpful fear-avoidance beliefs, as has been substantially demonstrated in non-specific low back pain [16].

A preliminary study enabled us to validate a French version [17] of an information booklet that draws on validated data from evidence-based medicine. The booklet was "*Le guide du coup de fouet cervical*," the French version of The Whiplash Book [18]. This study showed that fear-avoidance beliefs were considerably high in a population without neck problems who were working in hospitals. It also revealed that simply providing information could help lessen such beliefs.

Management of whiplash-associated disorders can make use of the recommendations published in the literature [18,19]. The priority is to rule out serious injury, and this usually entails a visit to an emergency medicine department after the accident. The decision to conduct radiographic assessment should be guided by validated clinical criteria as defined by the Canadian C-Spine Rule [20]. This involves a quick return to normal personal and work activity with the help of effective pain relief, in addition to the performance of specific mobilization exercises. This information can be found in the French National Authority For Health (HAS) guidelines [21] which state that "active mobilization techniques provide short-term benefit if implemented early." Most of these points were also stated by guidelines for the management of acute whiplash-associated disorders in 2014 [8]. The use of a neck collar should not be prescribed. Healthcare professionals must reassure and educate their patients that post-traumatic pain is normal and that they need to remain active and maintain physical activity in order to improve their prognosis [22].

As with back pain, healthcare professionals' fear-avoidance beliefs may influence their approach to management. Most practitioners adhere to the approach of encouraging an early return to activity rather than excessive rest, but patients do not follow these recommendations to any great extent [23,24,25]. Less than 10% of healthcare providers correctly identify initial neck pain intensity and disability as the two main predictors of poor recovery [26]. These unhelpful beliefs are about the cause and progression of symptoms, and they provoke confusion in patients' minds. It is vital to educate caregivers about the role of psychosocial factors such as beliefs about whiplash and fear-avoidance in recovery from whiplash, particularly when its pathophysiology is still unknown [14].

### Importance

Very few studies have evaluated the adherence of physicians or healthcare professionals to guidelines and to ways of changing their approach to treating whiplash [27,28,29].

### Goals of this investigation

Our study's main aim was to determine what fear-avoidance beliefs physicians have about the consequences of whiplash-associated disorders. The secondary aim was to measure the impact of a validated information booklet on emergency physicians' approach to management of whiplash-associated disorders.

## Materials and methods

### Study design and setting

This was a prospective cluster randomized controlled study involving a sample of emergency medicine physicians clustered in 13 hospitals. The study was granted approval by the local institutional review board, the *Comité de Protection des Personnes Sud Est VI* (IRB 00008526). Participants provided their written informed consent before participating in the study in accordance with the principles of the Helsinki Declaration. The study protocol was registered at ClinicalTrials. gov (NCT 03040245).

### Selection of participants

The workforce estimate recommended the inclusion of 24 physicians per randomization group, or about 4 physicians for 13 hospitals that said they would participate in the study. To take into account physician refusals, each hospital participating in the study was asked to include 10 physicians, which represented a potential number of 130 physicians for only 48 needed. In the end, by excluding hospitals that did not meet the target, we recruited 95 instead of 130 physicians. The 13 hospitals were randomized by considering a stratification according to the type of hospital. Of the 95 physicians included in these 13 hospitals, only 53 finally participated in the study, 5 more than expected. Cluster randomization was performed by computer and was conducted by a statistician working independent of the study. Seven hospitals were selected for the intervention group (reading The Whiplash Book), and 6 hospitals for the control group (no specific intervention).

### Interventions (The Whiplash Book)

The intervention group was given the French version of The Whiplash Book, an information booklet that has been validated in both English and French [17,30]. This is a specific document that makes use of validated data from evidence-based medicine and that recommends returning to activity early and mobilizing the neck in whiplash-associated disorders. It aims to reassure patients and emphasizes the importance of mobilizing the neck early and remaining active for better recovery. It provides illustrated guidance on the exercises to be performed.

### Control group (no Whiplash Book)

No particular intervention was conducted in the control group apart from giving them the two questionnaires.

### Methods and measurements

The primary endpoint was an assessment of fears and beliefs among the physicians tested by using a questionnaire, the Whiplash Belief Questionnaire (WBQ) [30], as previously validated in low back pain. The French WBQ version has already been used to validate The Whiplash Book and later the French version of the same booklet. The WBQ assesses fear-avoidance beliefs about the consequences of whiplash and has nine items. For each statement, the physician must express his or her level of agreement or disagreement, with a score that varies from 9 to 45. The higher the score, the stronger the beliefs [17,30].

Secondly, physicians' fear-avoidance beliefs were assessed by using a second questionnaire initially validated in non-specific back pain and adapted to neck conditions [17]. The Fear Avoidance Belief Questionnaire (FABQ) [31] in its validated French version [32] has two independent scales: the physical activity and work FABQ. The physical FABQ measures fear-avoidance beliefs relating to physical activity in general, namely the actions of daily life. It consists of four items with the score varying from 0 to 24. The work FABQ measures fear-avoidance beliefs in relation to work activities. It consists of seven items with the score varying from 0 to 42. The term "back pain" was replaced by "neck pain". Because the FABQ was designed for patients with pain, we modified the questionnaire for use with physicians [33]. Physicians had to declare their fear-avoidance beliefs concerning the patient’s injury. Approach to overall management in the emergency department meant the prescriptions and advice delivered to patients with whiplash-associated disorders. It was a secondary endpoint, evaluated in the form of a vignette before and after the intervention. All measurements were at the physician level. Physicians worked on a vignette or virtual clinical case without examining patients.

Vignettes are a valid way to measure clinical reasoning skills [34].The virtual clinical case described was a patient of working age. The clinical examination was reassuring, there were no neurological signs, and the vignette patient complained of neck pain. The case matched Grade 1 of the Quebec Task Force on Whiplash-Associated Disorders classification [7]. The closed-ended questions focused on clinical decision-making and treatment. Management was assessed on whether neck radiography was ordered and, if applicable, the length of time that pain relievers, non-steroidal anti-inflammatory drugs (NSAIDs), and muscle relaxants, a neck collar, and sick leave were prescribed. Physicians were also asked about any referral to physiotherapy (for how long and with what aim) as well as about the advice they delivered to patients with regard to maintaining physical activity and mobilizing the neck. A standard checklist of pre-defined answer options was used to assess the output based on guidelines. Two reviewers assessed the answers independently. In case of differences in scores, mutual agreement was achieved by discussion. The same vignette was presented for both intervention and control groups.

### Data collection

Once each of the department heads and the different physicians concerned had given their oral agreement to take part in the study after written information, an initial questionnaire folder (containing the WBQ and FABQ) was sent by email or by post to each of the physicians drawn by randomization. Upon inclusion, demographic data were collected from the physicians (gender, age, place of practice, and length of time in practice) as was any personal or family history of neck pain or whiplash-associated disorders. The physicians were also asked how often they encountered cases of whiplash-associated disorders.

Information about knowledge of whiplash-associated disorders was also sought. This information included continuing medical training or recent reading (within the previous 3 years), and in particular knowledge, of the different severity grades established by the Quebec Task Force on Whiplash-Associated Disorders classification [7], the radiologic recommendations of the Canadian C-Spine Rule [19], and finally the latest HAS recommendation on physical therapy in post-whiplash–associated disorders [21].

Once the first questionnaire folder had been completed, a second folder was then sent to all physicians participating in the study. The intervention group was instructed to complete the questionnaires at least 48 hours after the intervention, that is, after reading The Whiplash Book. The same items were included as in the initial folder, with an additional questionnaire enabling the intervention group to qualitatively assess the book based on a numerical scale from 0 to 10. If there was no response, reminders were sent by email, then by telephone, and finally by post. The delay between two questionnaires was identical for both intervention and control groups (at least 48 hours).

### Analysis

#### Sample size calculation

To assess the book’s impact on whiplash beliefs measured by means of WBQ, the sample size estimation was based on comparing the two study arms, with a two-sided type-I error of 5% and statistical power of 90%. According to the intra-class correlation coefficient (ICC for cluster randomized design) values reported usually in the literature [35,36] and to a database of ICCs at the University of Aberdeen [36], we estimated that ICC was not informative and was considered null in the context of this study. So, based on work proposed by Coudeyre *et al*. [16] and an effect size defined according to Cohen’s recommendations [37] [small (ES: 0.2), medium (ES: 0.5), and large (ES: 0.8, “grossly perceptible and therefore large”), 22 subjects would be needed per randomization group to highlight an ES = 1 (which corresponds to a minimal difference of about 5 points for a standard deviation of 4.78 to 5.37 concerning the WBQ) under the previously described assumptions. We eventually planned to include at least 24 participants per randomization group to take into account any physicians lost to follow-up.

#### Data analysis

Statistical analyses were performed with Stata v13 (StataCorp, College Station, TX, USA). The tests were two-sided, with α = 0.05. Continuous data are described with mean (±SD) or median (interquartile range [IQR]), according to statistical distribution, and categorical data with number (%). To evaluate the impact of a validated information book, the primary analysis was performed in line with Vickers and Altman [38] using baseline scores as independent variables. Multivariable analyses (random-effects regression models, linear for quantitative outcome and logistic for dichotomous dependent outcome) were performed after adjusting for parameters fixed according to univariate results and clinical relevance: baseline scores, age, gender, experience, and center (as a random effect to take into account cluster randomized design). For other parameters, randomized groups were compared with chi-square or Fisher's exact test for categorical variables and Student *t* test or Mann-Whitney test (normality assessed with the Shapiro-Wilk test and homoscedasticity with the Fisher-Snedecor test) for quantitative variables, as appropriate. These analyses were completed by intra-group comparisons performed with paired Student *t* test or Wilcoxon test for quantitative variables and Stuart-Maxwell test for categorical variables. P<0.05 was considered statistically significant.

## Results

### Characteristics of study participants

**Fig 1:**
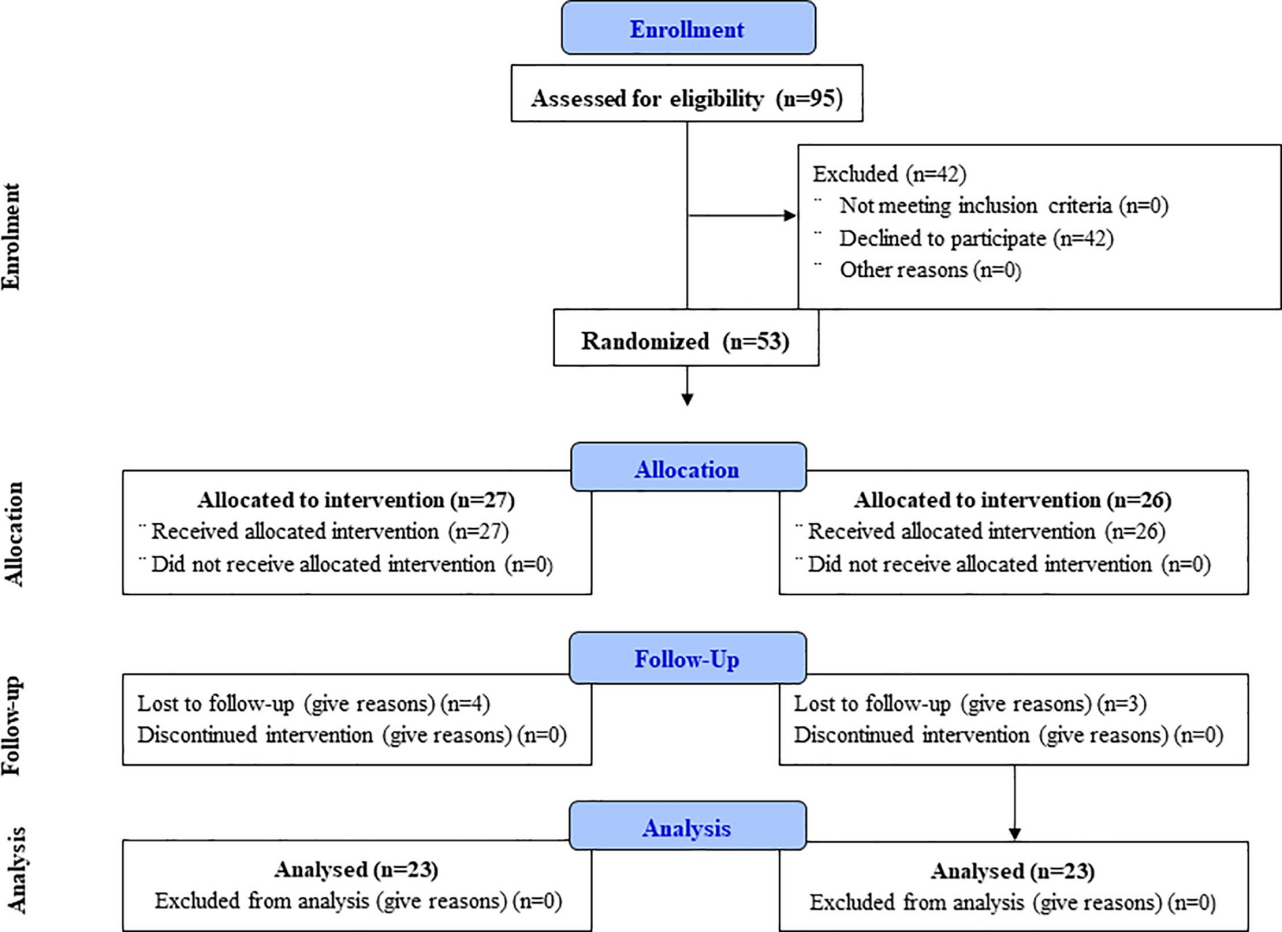
Flow chart.

In total, 95 physicians were invited to take part in the study; only 53 (56%) answered the first questionnaire, despite numerous reminders (n = 2.51±1.53). Of these 53 physicians, 46 answered the second questionnaire, after a mean of 1.35 reminders (±1.51). Final distribution between the control and intervention groups was 50% each, namely 23 physicians in each group, with a final response rate of 48.4% (Fig 1). The range of physicians in a hospital was two to eight.

The intervention and control groups (Table 1) were similar in most sociodemographic data except for gender and place of practice. The groups did not differ in history of neck pain and knowledge of whiplash-associated disorders. Mean age was 39.75 (±6.28) years. The mean length of time in practice was 10.71 (±6.22) years and mean length of time in the emergency department 9.28 (±5.39) years.

**Table 1:** Socio-demographic and professional data on study inclusion.

	Total (n = 53)	Control (n = 27)	Intervention (n = 26)	p
Gender male / female (%)	61/39	52/48	27/73	0.09
Age *(m* ± *SD)*	39.75 ± 6.28	39.22 ± 6.19	40.31 ± 6.46	0.53
Length of time in practice since thesis (years), (*m* ± *SD)*	10.71 ± 6.22	10.33 ± 5.94	11.13 ± 6.62	0.65
Length of time in emergency practice (years), *(m* ± *SD)*	9.28 ± 5.39	9.08 ± 5.34	9.48 ± 5.54	0.79
FABQ *(m* ± *SD)*	** **	** **	** **	
FABQ physical activity [0–24]	11.45 ± 4.73	10.67 ± 4.80	12.27 ± 4.60	0.22
FABQ work [0–42]	13.85 ± 6.70	13.37 ± 6.91	14.35 ± 6.58	0.60
WBQ *(m ± SD)* [9–45]	19.09 ± 4.06	19.67 ± 4.25	18.50 ± 3.84	0.30

m ± SD **=** mean ± standard deviation; FABQ = Fear Avoidance Belief questionnaire; WBQ = Whiplash Belief Questionnaire

It was not unusual to encounter whiplash-associated disorders in the emergency department, given that 74% of the physicians considered such cases to be common or very common, whereas only 26% deemed them uncommon or rare. The emergency physicians' knowledge of whiplash-associated disorders appeared to be poor (Table 2). Overall, 10% of physicians were aware of the Quebec Task Force on Whiplash-Associated Disorders classification, Canadian C-Spine Rule, and HAS recommendations. Post-university training in whiplash-associated disorders seemed almost non-existent (90% of physicians).

**Table 2:** Knowledge and education on whiplash injury.

	Baseline			Study Endpoints		
	Total (n = 53)	Control (n = 27)	Intervention (n = 26)	Control (n = 23)	Intervention (n = 23)	p
Recent education (<3 years) focused on cervicalgia or whiplash, n (%)	5 (9.4)	2 (7.4)	3 (11.5)	0 (0.0)	1 (4.4)	1.00
Recent reading of articles focused on cervicalgia or whiplash, n (%)	19 (35.9)	13 (48.2)	6 (23.1)	6 (26.1)	2 (8.7)	0.24
Awareness of Quebec Task Force on Whiplash-Associated Disorders classification, n (%)	4 (7.7)	3 (11.1)	1 (4.0)	7 (30.4)	3 (13.0)	0.15
Awareness of Canadian C-spine Rule, n (%)	6 (11.3)	4 (14.8)	2 (7.7)	7 (30.4)	3 (13.0)	0.16
Awareness of HAS recommendations, n (%)	3 (5.7)	0 (0.0)	3 (11.5)	4 (17.4)	4 (17.4)	1.00

HAS-Haute Autorité de Santé (French National Authority for Health)

### Main results

On inclusion, the groups were similar in terms of fear-avoidance beliefs (Table 1) and approach to diagnosis and treatment (Table 3). Mean fear-avoidance beliefs scores on inclusion were high: WBQ = 19.09 (±4.06); physical activity FABQ = 11.45 (±4.73); and work FABQ = 13.85 (±6.70) (Table 1). Almost one-quarter of the physicians had a physical activity FABQ score > 14, the threshold value above which fear-avoidance beliefs levels are considered very high [31].

**Table 3:** Diagnostic and therapeutic management of whiplash in emergency departments.

	Baseline			Study Endpoints		
	Total (n = 53)	Control (n = 27)	Intervention (n = 26)	Control (n = 23)	Intervention (n = 23)	p
Cervical spine radiology, n (%)	53 (100.0)	27 (100.0)	26 (100.0)	21 (91.3)	20 (87.0)	0.64
Prescription of Step 1analgesics, n (%)	53 (100.0)	27 (100.0)	26 (100.0)	23 (10.00)	23 (100.0)	1.00
If yes, duration of prescription, days, n (%)						0.90
<3	1 (1.9)	1 (3.7)	0 (0)	3 (13.1)	2 (8.7)	
3 to 8	47 (88.7)	24 (88.9)	23 (88.5)	19 (82.6)	20 (87.0)	
>8	5 (9.4)	2 (7.4)	3 (11.5)	1 (4.3)	1 (4.3)	
Prescription of NSAIDs, n (%)	41 (77.3)	20 (74.0)	21 (80.8)	18 (78.3)	17 (73.9)	0.73
If yes, duration of prescription, days, n (%)						0.41
<3	8 (19.5)	5 (25.0)	3 (14.3)	6 (33.3)	8 (47.1)	
3 to 8	33 (80.5)	15 (75.0)	18 (85.7)	12 (66.7)	9 (52.9)	
>8	0 (0.0)	0 (0.0)	0 (0.0)	0 (0.0)	0 (0.0)	
Prescription of myorelaxant agents, n (%)	41 (77.3)	20 (74.0)	21 (80.8)	16 (69.6)	16 (69.6)	1.00
If yes, duration of prescription, days, n (%)						0.72
<3	10 (24.4)	4 (20.0)	6 (28.6)	5 (31.3)	7 (43.7)	
3 to 8	30 (73.2)	15 (75.0)	15 (71.4)	10 (62.5)	9 (56.3)	
>8	1 (2.4)	1 (5.0)	0 (0.0)	1 (6.2)	0 (0.0)	

NSAID = nonsteroidal anti-inflammatory drug

**Fig 2:**
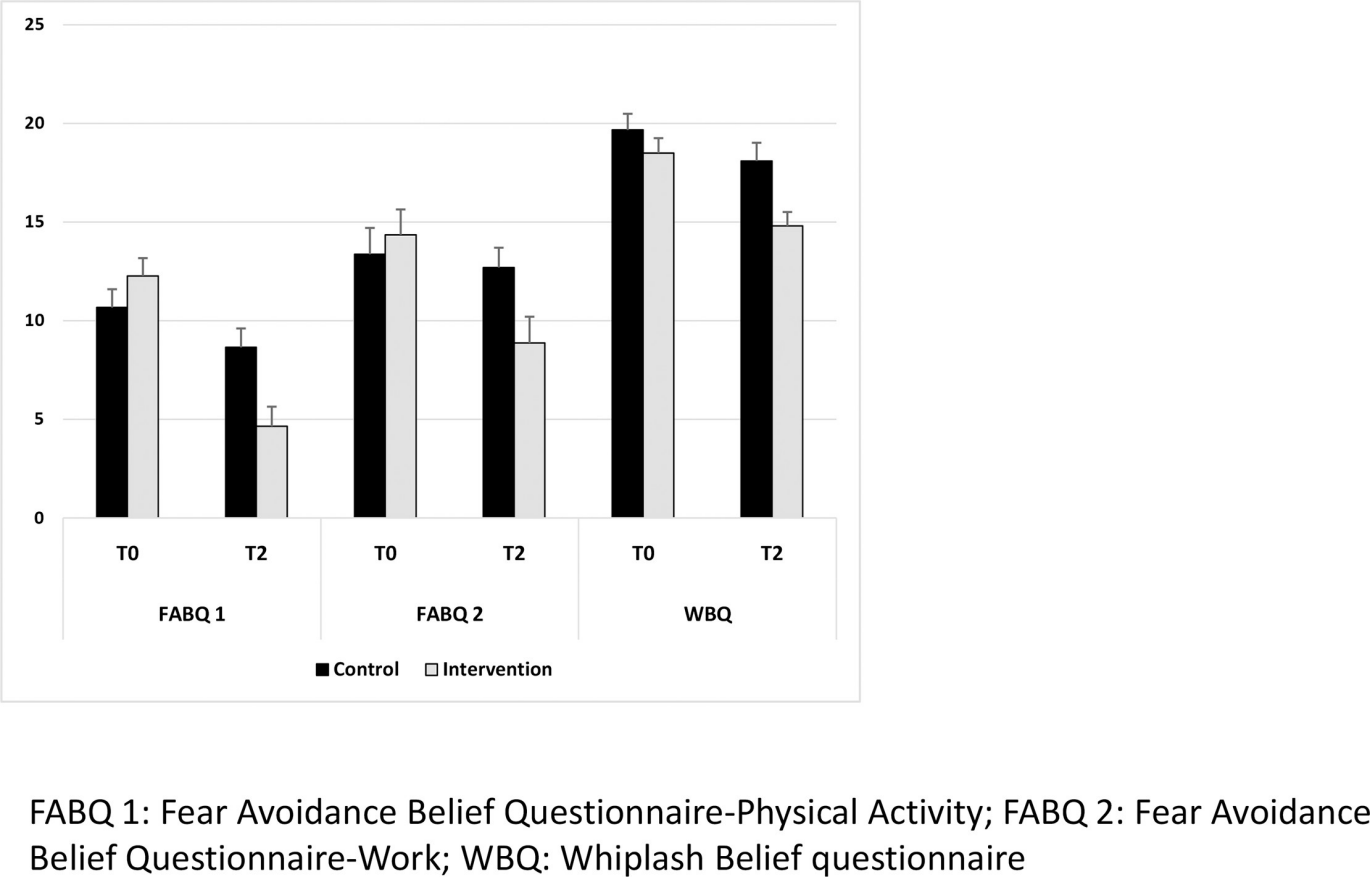
Follow up data.

Considering the primary endpoint, intra-group analysis revealed a significant decrease in WBQ score in the intervention group (18.4±3.8 vs. 14.8±3.4, p < 0.001), with no significant difference in the control group (19.5±3.7 vs. 18.2±4.4, p = 0.10), so the WBQ score decreased more in the intervention than control group (p = 0.01; ES = 0.86 [0.25; 1.46]) (Fig 2). The ICC estimated from the random-effects model was <0.001. Additional analysis using a multivariable regression model revealed a difference between groups of -3.10 (-5.33; -0.88) (p = 0.006). The analysis of percentage change in WBQ scores confirmed that improvement in fear-avoidance beliefs was greater in the intervention than control group (-20 [-32; -6] vs. -6 [-16; 9]; p = 0.06) (Table 1).

Intra-group analysis showed a significant decrease in physical activity FABQ score in both the intervention (p<0.001) and control (p = 0.02) groups, with FABQ score decreasing more in the intervention than control group (p = 0.001; ES = 0.85 [0.24; 1.45]) (Fig 2). Additional analysis with a multivariate regression model revealed a regression coefficient of -4.98 (-7.36; -2.61) (p<0.001). Analysis of variation in physical activity FABQ scores confirmed that improvement in fear-avoidance beliefs was greater in the intervention than control group (-70 [-86; -50] *vs*. -15 [-40; 11]; p<0.001). Similar results were observed for the work FABQ. The work FABQ score significantly decreased in the intervention group (p = 0.01), with no significant difference in the control group (p = 0.53). The work FABQ score decreased more in the intervention than control group (p = 0.02; ES = 0.68 [0.08; 1.27]) (Fig 2). Additional analysis with a multivariate regression model revealed a regression coefficient of -4.12 (-7.41; -0.84) (p = 0.01). Analysis of variation in the work FABQ scores confirmed that improvement in fear-avoidance beliefs was greater in the intervention than control group (-40 [-71; 0] *vs*. 0 [-31; 50]; p = 0.02) (Table 1). Variation in the FABQ score for fears and beliefs was not significantly correlated with age and length of time in practice, given that the respective correlation coefficients were low, at 0.12 (p = 0.43) and -0.20 (p = 0.18), respectively. Similar correlation coefficients were found for the WBQ score, namely, 0.20 (p = 0.17) and -0.13 (p = 0.38), respectively.

### Pharmacological and non-pharmacological treatments

These results were collected from the responses to the vignette by physicians. Emergency radiography was indicated in all cases, with a high level of pharmacological treatment prescribed. All the physicians prescribed simple analgesics for 3 to 8 days in 90% of cases, with 8/10 physicians prescribing non-steroidal anti-inflammatory drugs and muscle relaxants (73%) (Table 3).

It was very common to use neck collars for immobilization (86% of physicians), and they were used for relatively long periods. However, sessions with a physical therapist were not often prescribed (15%), and in half of the cases, these sessions were scheduled after the 8th day (Table 4).

**Table 4:** Non-pharmacological management.

	Baseline			Study Endpoints		
	Total (n = 53)	Control (n = 27)	Intervention (n = 26)	Control (n = 23)	Intervention (n = 23)	p
Prescription of neck brace, n (%)	46 (86.8)	24 (88.9)	22 (84.6)	19 (82.6)	18 (78.3)	0.72
If yes, duration of prescription, n (%)						0.047
<3	2 (4.4)	2 (8.7)	0 (0.0)	4 (21.0)	11 (61.1)	
3 to 8	29 (64.4)	13 (56.5)	16 (72.7)	9 (47.4)	5 (27.8)	
>8	14 (31.2)	8 (35.8)	6 (27.3)	6 (31.6)	2 (11.1)	
Prescription of physiotherapy sessions, n (%)	8 (15.1)	4 (14.8)	4 (15.4)	7 (30.4)	5 (21.7)	0.50
If yes, when?						0.42
Day 0	2 (25.0)	0 (0.0)	2 (50.0)	1 (14.3)	2 (33.3)	
Day 3	0 (0.0)	0 (0.0)	0 (0.0)	2 (28.6)	3 (50.0)	
3 to 8 days	2 (25.0)	1 (25.0)	1 (25.0)	1 (14.3)	1 (16.7)	
>8 days	4 (50.0)	3 (75.0)	1 (25.0)	3 (42.8)	0 (0.0)	
With which intention? n (%)						
Analgesic (1)	2 (25.0)	1 (25.0)	1 (25.0)	0 (0.0)	1 (16.7)	0.42
Gain of motility (2)	0 (0.0)	0 (0.0)	0 (0.0)	2 (28.6)	1 (16.7)	
Muscle reinforcement (3)	2 (25.0)	1 (25.0)	1 (25.0)	1 (14.3)	0 (0.0)	
1+2	0 (0.0)	0 (0.0)	0 (0.0)	1 (14.3)	0 (0.0)	
2+3	3 (37.5)	1 (25.0)	2 (50.0)	0 (0.0)	2 (33.2)	
1+2+3	1 (12.5)	1 (25.0)	0 (0.0)	3 (42.8)	1 (16.7)	
Other	0 (0.0)	0 (0.0)	0 (0.0)	0 (0.0)	1 (16.7)	
Work cessation yes/no, n (%)	39 (78.0)	20 (76.9)	19 (79.1)	13 (56.5)	7 (30.4)	0.07
If yes, duration, days, n (%)						0.27
<3	12 (30.8)	6 (30.0)	6 (31.6)	5 (41.7)	5 (83.3)	
3 to 8	25 (64.1)	13 (65.0)	12 (63.1)	5 (41.7)	1 (16.7)	
>8	2 (5.1)	1 (5.0)	1 (5.3)	2 (16.6)	0 (0.0)	
Advice pertaining to activity, n (%)						
Bed rest in the event of pain (1)	0 (0.0)	0 (0.0)	0 (0.0)	0 (0.0)	0 (0.0)	0.25
Rest at home in the event of pain (2)	10 (18.9)	3 (11.1)	7 (26.9)	3 (13.0)	1 (4.4)	
Maximum tolerated activity (3)	24 (45.3)	13 (48.1)	11 (42.3)	10 (43.5)	16 (69.5)	
Normal activity (4)	17 (32.0)	10 (37.1)	7 (26.9)	9 (39.1)	6 (26.1)	
(2) and (4)	1 (1.9)	0 (0.0)	1 (3.8)	0 (0.0)	0 (0.0)	
(3) and (4)	1 (1.9)	1 (3.7)	0 (0.0)	1 (4.4)	0 (0.0)	
Advice pertaining to mobilization, n (%)						
Immobilization of the neck in the event of pain (1)	13 (25.0)	7 (25.9)	6 (24.0)	5 (21.7)	3 (13.0)	0.69
Cautious mobilization of the neck in the event of pain (2)	15 (28.9)	6 (22.2)	9 (36.0)	4 (17.4)	3 (13.0)	
Mobilization below pain threshold (3)	15 (28.89)	8 (29.6)	7 (28.0)	10 (43.5)	10 (43.5)	
Maximum tolerated mobilization (4)	4 (7.7)	1 (3.7)	3 (12.0)	3 (13.0)	6 (26.1)	
(1) and (2)	0 (0.0)	0 (0.0)	0 (0.0)	1 (4.4)	0 (0.0)	
(1) and (3)	2 (3.8)	2 (7.4)	0 (0.0)	0 (0.0)	0 (0.0)	
(2) and (3)	1 (1.9)	1 (3.7)	0 (0.0)	0 (0.0)	1 (4.4)	
(1) and (2) and (3)	2 (3.8)	2 (7.4)	0 (0.0)	0 (0.0)	0 (0.0)	

Sick leave was prescribed by more than three-quarters of the physicians, whereas nearly 80% of the emergency physicians advised that the patient maintain the maximum tolerated level of activity, or even normal activity. However, more than one in two physicians recommended that the neck be kept immobilized or be mobilized with caution, as long as the pain lasted. The emergency physicians (96%) did not take advantage of any patient-friendly information handouts or other materials to back up their advice (Table 4).

In intra-group analysis, reading the French version of The Whiplash Book changed the approach to management in several areas. For example, we found a significant decrease in the prescription of sick leave (p = 0.002) as well as a non-significant reduction in duration of immobilization (60% for < 3 days) but no change in frequency of prescription. Pharmacologically, only the duration of NSAID prescriptions was significantly reduced (p = 0.01), with no significant change in the control group. Of note, 3/10 (20%) physicians sought information about whiplash-associated disorders between delivery of the two questionnaires, predominantly in the control group.

The physicians had a very favorable opinion of The Whiplash Book, which received a mean score of 7.45/10 (±1.34). Nearly 75% stated that they had gained very useful information, and 8 in 10 physicians said that they agreed with the entire book. Overall, 95% of the physicians thought that the book could help patients, and that they would give it to those with whiplash-associated disorders. The book’s length appeared to have posed a problem for 74% of physicians.

## Discussion

Our study showed that the current practice among emergency physicians from a French region did not comply with good practice guidelines for whiplash and that an information campaign via an information book intended for patients might lessen their fear-avoidance beliefs. Our results agree with data published in the literature, although literature focused on this topic appeared limited [27,28,29]. After healthcare providers undertook online learning over 3 years, 57.2% of participants improved their knowledge regarding treatment by more than 20% as compared with the beginning of the study. Lower baseline knowledge was associated with greater improvement [27]. As described in Statistical section, sample size estimation was based on an ES defined according to Cohen’s recommendations. For a statistical power of 90%, 22 participants per group were needed to highlight an ES = 1. For a statistical power of 80%, this sample size allows for showing an ES = 0.85. As presented in the Results section, the effect size concerning the primary endpoint was 0.86 [0.25; 1.46]. So, despite the relatively limited sample size, the statistical power seems totally robust and satisfactory (>80%) to support our conclusions.

Moreover, a recent cluster randomized trial in the United Kingdom justified the use of The Whiplash Book as an education booklet designed in our study for clinicians [39]. It has now been clearly established that plain radiography should not be systematically performed in all cases. The Canadian C-Spine Rule, among others, clearly identifies which patients require radiography, yet only 1 in 10 physicians seemed to be familiar with the rule. Our study showed that simply reading the booklet did not result in a significant decrease in number of plain radiographs performed. The fear of missing a fracture and of being held responsible for it appeared to be a strong motivator for requesting radiography. The Canadian C-Spine Rule offers simple guidelines for physicians worried about missing a serious disorder, a concern that is not backed up by epidemiological data.

As for Canadian practitioners [25], we found a high level of drug prescription, such as analgesics, NSAIDs, and muscle relaxants, despite a lack of evidence on their efficacy in acute post-whiplash–associated disorders. Reading the whiplash guide could help improve this practice.

Practice guidelines recommend an early return to physical activity, along with any rehabilitation techniques that include early mobilization [1,20,40]. However, we have shown that only a few emergency physicians spontaneously thought of prescribing physical therapy sessions. Also, if they did so, all too often, such sessions were prescribed some time following the accident. Reading the booklet and improving fear-avoidance beliefs were not enough to change this practice. This situation may be due to emergency physicians generally not being in the habit of prescribing sessions of physical therapy, entrusting this task to family physicians instead.

These deviations from the guidelines and factual data are probably caused by a lack of information and post-university training provided to physicians, particularly when data and pre-existing classifications are concerned. Only a few emergency physicians appeared to be aware of the Canadian C-Spine Rule, Quebec Task Force on Whiplash-Associated Disorders classification, or HAS guidelines.

Physicians' non-adherence to the guidelines may be due to several factors. First, these guidelines may not be well known, as confirmed by our study data. Physicians may be in complete disagreement with the guidelines, considering them to be ill-suited. Another factor may be the risk of legal issues, particularly the fear of failing to spot a severe cervical spine disorder. Finally, fear-avoidance beliefs among practitioners are another factor accounting for poor compliance with the guidelines. In line with the literature dealing with low back pain [33], our study showed that these fear-avoidance beliefs stem more from personal feelings linked to the physician’s own past or those close to them rather than from data derived from evidence-based medicine.

Numerous studies conducted in the setting of non-specific low back pain have demonstrated the impact of physicians' fear-avoidance beliefs on their approach to treatment [41] as well as the utility of an information campaign for both healthcare professionals and the general population on the natural history of non-specific low back pain. The literature has also provided data on this topic in the whiplash setting [42]. What patients expect from their treatment relative to what they consider to be best may affect healthcare providers' treatment strategy. The medical professionals that the patient sees after the initial treatment phase may also provide the patient with contradictory information, thereby possibly creating risk factors for the pain becoming chronic [25]. The Whiplash Book, which was initially intended for patients, does not comprise all the treatment guidelines meant for doctors. This may explain the "only" partial change in their approach to management. As a result, lowering fears and beliefs alone did not allow for fundamental changes in management of whiplash in emergency departments. However, this observation confirms the usefulness of an information booklet for modifying fear-avoidance beliefs among physicians, a concept that was already demonstrated in individuals without neck conditions [17].

The recommendations made by the physicians for the vignette patient could nonetheless not be compared, given that several physicians provided multiple answers, thereby resulting in a variety of possible responses. Yet, paradoxically, the advice given was consistent with the guidelines regarding maintaining the maximum tolerated level of physical activity with whiplash-associated disorders.

### Limitations

A retrospective study based on patients' medical file analysis might have been more realistic for assessing physicians' actual approach to treatment. Indeed, the study we conducted investigated intended treatments, these being at times different from actual practice.

Therefore, The Whiplash Book seems to be one of the means available for modifying fear-avoidance beliefs and modifying, at least to some extent, physicians' approach to management. Moreover, reading the booklet provided physicians with the opportunity to gain further knowledge, which 75% of them considered very useful for their practice, whereas 95% of emergency physicians thought giving the booklet to their patients worthwhile. It may be a good idea to disseminate the booklet on a large scale, to contribute to changing practices among all healthcare professionals and as editorial material to back up their advice to patients, given that it has been shown to lower fear-avoidance beliefs in the general population [43].

However, our analyses did not estimate the role of barriers and facilitators to compliance with guidelines. It is widely reported that dissemination of clinical guidelines alone is unlikely to change health professional’s knowledge or practice [44]. Rather, the literature suggests that targeted and active implementation strategies need to be used and barriers to implementation identified to change professional knowledge and practice. Thus, professional background and the beliefs system of the health professional are potential factors that may arise as barriers to implementation [45]. Low baseline knowledge could significantly contribute to the size of a learning effect. Therefore, concerning whiplash guidelines, a prospective cohort study suggested that by measuring baseline knowledge with a questionnaire, implementation costs could be saved by directing education at practitioners with low knowledge rather than attempting to educate all practitioners [28].

The clustered analysis was dealt with in a statistically sophisticated way, but there is still a risk of institutional practice biases or education. The low response rate also could introduce bias, with only physicians more likely to be receptive to reading a pamphlet responding. The early duration of outcome testing is also a major limitation in that whether the results translate to any changes in beliefs long term remains unknown.

In summary, reading a validated French version of the information booklet, The Whiplash Book, initially intended for patients, was shown to lower fear-avoidance beliefs among emergency physicians in the French region. However, the early duration of outcome testing is also a limitation in that whether the results translate to any changes in beliefs long term remains unknown.

That the emergency physicians did not follow the guidelines for whiplash in clinical practice was probably due to a lack of information. As with non-specific low back pain, developing a policy of delivering information on whiplash to both healthcare professionals and the general population undoubtedly has benefits. Ultimately, whether this intervention translates to clinical practice changes remains unknown, and the vignette analysis offers only a small signal of efficacy amidst many variables that did not change. Investigating different strategies for putting the recommendations published in the literature into clinical practice may be worthwhile.

The Whiplash Book could be a part of this strategy. The Whiplash Book was favorably received among the emergency physicians, who currently do not have any informative material to distribute to patients with whiplash-associated disorders in order to back up their advice. However, targeted implementation strategies should be used across all emergency departments and more generally with all healthcare professionals who may exert a favorable impact on the repercussions of whiplash-associated disorders. A larger-scale study including evaluation of barriers and facilitators to implementation guidelines with a measure of baseline knowledge appears warranted to confirm these encouraging yet preliminary data.

## Supporting information

S1 Checklist CONSORT 2010 checklist of information to include when reporting a randomised trial*. (DOC)

S1 Protocole (DOC)
