# Supplementary material for: Measuring the impact of the French version of The Whiplash Book on both treatment approach and fear-avoidance beliefs among emergency physicians. A cluster randomized controlled trial
Source: PLoS One. 2020 Mar 18;15(3):e0229849. doi: 10.1371/journal.pone.0229849 (PMC7080520; doi:10.1371/journal.pone.0229849)
Supplement: S1 Protocole — (DOC) [file pone.0229849.s002.doc]

**CENTRE HOSPITALIER UNIVERSITAIRE DE CLERMONT-FERRAND**

**CLINICAL TRIAL PROTOCOL**

**Measuring the impact of the French version of the Whiplash Book on both treatment approach and fears and beliefs among emergency physicians. A randomized controlled trial.**

**Sponsor :** CHU de Clermont-Ferrand

**Principal Investigator :** Pr Emmanuel Coudeyre

Service de Médecine Physique et de Réadaptation

Hôpital Gabriel Montpied - CHU de Clermont-Ferrand

58 rue Montalembert

63003 Clermont-Ferrand cedex 1

**Study setting :** Service de Médecine Physique et de Réadaptation

Hôpital Gabriel Montpied - CHU de Clermont-Ferrand

58 rue Montalembert

63003 Clermont-Ferrand cedex 1

**TABLE OF CONTENTS**

**Page**

**Synopsis** **3**

**1- GENERAL INFORMATION 4**

**2- STUDY RATIONNALE 7**

2.1- Background 7

2.2- Practice guidelines 8

2.3- Impact of information leaflets 8

**3- STUDY OBJECTIVE 8**

**4- EXPERIMENTAL PLAN 8**

4.1- Leaflet elaboration………………………………………. ……………………………………………………8

4.2- Randomized controlled study ………………………………………………………………………………..9

4.2.1.-Expérimental Plan…………………………………………………………………………………..9

4.2.2- Subjects selection …………………………………………………………………………………..9

4.2.3- Randomization……………………………………………………………………………………..9

4.2.4- Outcomes ………………………………………………………………………………………….9

4.3- Design of trial to be conducted………………………………………………………………………….......10

4.3.1- For the physician…………………………………………………………………………………..10

**5 STATISTICS 11**

5.1- Subjects number 11

5.2- Data Analysis 11

**6- STUDY FEASIBILITY AND EXPECTED RESULTS 12**

6.1- Study feasibility 12

6.2- Expected results 12

**7 ETHICS AND LEGAL FACTS 13**

7.1- Usual care 13

**7.2- Registration 13**

[**7.3-**](#__RefHeading___Toc157336626) **Source documents 13**

7.4- Quality insurance 14

7.5 Quality management 14

[**7.6-**](#__RefHeading___Toc157336627) **Physician information 14**

[**7.7- CRF**](#__RefHeading___Toc157336628) **report 14**

**7.8- Confidentiality 15**

[**7.9-**](#__RefHeading___Toc157336631) **Protocol modifications 15**

7.10- Trial extension 15

7.11- Archiving 16

[**7.12- Final report**](#__RefHeading___Toc157336632) **16**

7.13- Financing 16

[**7.14- Publications and**](#__RefHeading___Toc157336633) **date properties 16**

**8- REFERENCES…………………………………………………………………………...17**

**SYNOPSIS**

| **Title** | Measuring the impact of the French version of the Whiplash Book on both treatment approach and fears and beliefs among emergency physicians. A randomized controlled trial. |
| --- | --- |
| **Primary Investigator** | Pr Emmanuel Coudeyre |
| **Investigation Center** | CHU Clermont-Ferrand |
| **Objectives** | **Primary objective**  To determine what fears and beliefs physicians have about the consequences of whiplash. To measure the impact of a validated information booklet on emergency physicians' approach to management following a whiplash injury. |
| **Expérimental plan** | Randomized controlled study |
| **Physician number** | To assess the booklet's impact on whiplash beliefs measured by means of Whiplash belief questionnaire (WBQ), the sample size estimation was based on comparing the two study arms, with a two-sided Type-I error of 5% and statistical power of 90%. 22 subjects would be needed per randomization group in order to highlight an effect-size equaling 1 (which corresponds to a minimal difference of about 5 points for a standard-deviation between 4.78 and 5.37 concerning WBQ) under the previously described assumptions. It was eventually planned to include 24 subjects per randomization group in order to take into account any physician lost to follow up. |
| **Physician follow up** | Between 48 hours and one month |
| **Study Duration** | 4 months |
| **Study flow** | - V0 inclusion - Intervention (information leaflet) - Follow-up at least 48 hours |
| **Inclusion and non inclusion criteria** | The study was conducted involving a sample of 95 emergency physicians working in different hospital facilities in the Auvergne region, France. Hospitals were split into two groups: the six hospitals with the highest volumes, and the other seven hospitals, considered as local hospitals. |
| **Outcomes** | **Primary outcome** :  The primary endpoint was an assessment of fears and beliefs in the sample of physicians tested using a questionnaire, the WBQ, as previously validated in low back pain.  **Secondary outcomes** :  Secondarily, the physicians' fears and beliefs were also assessed using a second questionnaire initially validated in non-specific back pain and adapted to neck conditions. The FABQ (Fear Avoidance Belief questionnaire) in its validated French version comprises two independent scales: the physical activity FABQ and work FABQ. Approach to overall management in the emergency department meant the prescriptions and advice delivered to patients who suffer from whiplash injury. It was a secondary endpoint, evaluated in the form of a clinical case. |

Key words: Whiplash injury; Pamphlet; Beliefs; Education; Fear-avoidance.

.

**1- GENERAL INFORMATIONS**

**RESEARCH TITLE**: Measuring the impact of the French version of the Whiplash Book on both treatment approach and fears and beliefs among emergency physicians. A randomized controlled trial.

**PROMOTION**

CHU de Clermont-Ferrand

**Délégation à la Recherche Clinique & à l’Innovation**

**Direction Générale Adjointe**

**Villa annexe IFSI**

**58 rue Montalembert**

**63003 Clermont-Ferrand Cedex 1**

Tél : 04.73.751.195 / Fax : 04.73.754.730

**PRIMARY INVESTIGATOR**

Pr Emmanuel Coudeyre

Service de Médecine Physique et de Réadaptation

Hôpital GABRIEL MONTPIED - CHU de Clermont-Ferrand

58 rue Montalembert

63003 Clermont-Ferrand cedex 1

**CO-INVESTIGATORS**

Pr Jeannot Schmidt

Service des Urgences

Hôpital Gabriel Montpied - CHU de Clermont-Ferrand

58 rue Montalembert

63003 Clermont-Ferrand cedex 1

Dr Christophe Perrier

Service des Urgences

Hôpital Gabriel Montpied - CHU de Clermont-Ferrand

58 rue Montalembert

63003 Clermont-Ferrand cedex 1

Dr Stéphane Poizat

Service de Médecine Physique et Réadaptation

CHU Clermont-Ferrand, Hôpital Nord

61 route de Chateaugay, BP 30056

63118 Cébazat

Pr Laurent Gerbaud

Service de Santé Publique

Hôpital Gabriel Montpied - CHU de Clermont-Ferrand

58 rue Montalembert

63003 Clermont-Ferrand cedex 1

Dr Candy Auclair

Service de Santé Publique

Hôpital Gabriel Montpied - CHU de Clermont-Ferrand

58 rue Montalembert

63003 Clermont-Ferrand cedex 1

**PARTNERS**

Bruno Pereira, Biostatistician

Direction de la Recherche Clinique

Hôpital Gabriel Montpied - CHU de Clermont-Ferrand

58 rue Montalembert

63003 Clermont-Ferrand cedex 1

**STUDY LOCATION**

Service Médecine Physique et Réadaptation

Hôpital Gabriel Montpied - CHU de Clermont-Ferrand

58 rue Montalembert

63003 Clermont-Ferrand cedex 1

**DATA ANALYSIS**

Department of Physical Medicine and Rehabilitation, Clermont-Ferrand University Hospital, in collaboration with DRCI (Innovation and Clinical Research).

**STUDY PLANNING**

Submission to technical committee May 2015

Submission to ethical committee May 2015

Inclusion time: 3 months (September 2015 to November 2015)

Participation duration for each patient: 1 hour (inclusion visit) + 1 hour (follow-up questionnaires)

End of the study: December 2015

Data analysis and final report: February 2016

# 2- STUDY RATIONNALE

## 2.1 Background

Whiplash is defined as minor indirect trauma to the cervical spine following a collision from behind at low speed or when stopped. It is an acceleration-deceleration mechanism of energy transfer to the neck that occurs while driving in a town or city . The main obstacle to managing whiplash is that it is first and foremost a lesion mechanism and not a unique pathologic entity. This transfer of energy leads to bone or soft tissue lesions that can result in a variety of clinical manifestations.

Whiplash trauma and the resulting whiplash associated disorders have been the subject of much attention in the scientific literature and remain a major public health problem .

The most commonly encountered symptoms are neck pain, headache, low back pain, shoulder pain, as well as visual impairment . Patients can be categorized by lesion grade according to the Quebec task force classification . Following a whiplash injury, 44% of patients are asymptomatic at 1 month following the trauma, 65% at 12 months, and 75% at 5 years . However, it is not unusual for neck pain to become chronic, and when this occurs, there may be serious consequences on a social, professional and financial level.

Of the demographic and accidentological factors most often associated with chronic neck pain, there are the following parameters: initial pain intensity, a high number of initial symptoms, anxiety, and severity of the injury as perceived by the patient . Even though the role of the initial episode should not be ignored, progression to chronic pain is probably multifactorial and, like non-specific low back pain, psycho-social factors, and in particular patient fears and beliefs , seem to play an relevant role, as do environmental factors.

2.2 Practice guidelines

Management following whiplash injury can make use of the recommendations as published in the literature . The decision to conduct radiographic assessment should be guided by validated clinical criteria as defined by the Canadian C-Spine Rule . This involves a quick return to normal personal and work activity with the help of effective pain relief, in addition to the performance of specific mobilization exercises. This information can be found in the French National Authority For Health (HAS) guidelines which state that "active mobilization techniques provide short-term benefit if implemented early ." Most of these points were also stated by the South Australian Centre for Trauma and Injury Recovery in 2008 . Healthcare professionals must reassure and educate their patients that post-traumatic pain is normal, and that they need to remain active and maintain physical activity in order to improve their prognosis.

The professional consensus is that it is useful to provide targeted information at an early stage in whiplash settings in order to reduce mistaken fears and beliefs, as has been substantially demonstrated in non-specific low back pain.

As with back pain ,, healthcare professionals' fears and beliefs may influence their approach to management. Whereas most practitioners adhere to the approach of encouraging an early return to activity rather than excessive rest, patients do not follow these recommendations to any great extent . Less than 10% of healthcare providers correctly identify initial neck pain intensity and disability as the two main predictive factors of poor recovery . These mistaken beliefs are about the cause and progression of symptoms, and they provoke confusion in patients' minds It is vital to educate care-givers about the relevance of patients' fears regarding the prognosis of the initial trauma lesion, particularly when its pathophysiology is still unknown .

2.3 Interest to use a standardized booklet

A preliminary study enabled us to validate a French version of an information booklet that draws on validated data from evidence-based medicine. The booklet was "*Le guide du coup de fouet cervical*," the French version of The Whiplash Book . This study showed that fears and beliefs were considerably high in a population without neck problems who were working in hospitals. It also revealed that simply providing information could help lessen them.

**3- STUDY OBJECTIVE**

Our study's main aim was to determine what fears and beliefs physicians have about the consequences of whiplash. The secondary aim was to measure the impact of a validated information booklet on emergency physicians' approach to management following a whiplash injury.

**4- EXPERIMENTAL PLAN**

This was a prospective randomized controlled study conducted involving a sample of emergency medicine doctors.

4.1 Intervention

The intervention group was given the French version of "The Whiplash Book," an information booklet that has been validated in both English and French . This is a specific document that makes use of validated data from evidence-based medicine and that recommends returning to activity early and mobilizing the neck following whiplash injury. It aims to reassure patients and emphasizes the importance of mobilizing the neck early and of remaining active for better recovery. It provides illustrated guidance on the exercises to be performed. No particular intervention was conducted in the control group apart from giving them the two questionnaires.

4.2 Randomized controlled study

4.2.1 Experimental plan and justification

We will implement a multicenter randomized controlled trial comparing:

1) Single educational support in the form of an information booklet.

2) No particular intervention was conducted in the control group apart from giving the questionnaires.

The randomized controlled trial is the gold standard for therapeutic evaluation. Physicians randomized to the control group will not have specific information.

Physician will be blinded about the hypothesis. They will be informed that the objective of this study will be to evaluate an information booklet. However, the content of each take charge and the hypothesis of the study they will not be detailed. This strategy should limit bias, particularly the risk of bias assessment. This method is ethically acceptable because physician will be informed that for scientific reasons we cannot explain all the hypotheses of the study. Physician will all be informed at the end of the study hypotheses and results of the study if they wish.

4.2.2 Subjects selection

## 4.2.3 Outcomes

a)- Main outcome

The primary endpoint was an assessment of fears and beliefs in the sample of physicians tested using a questionnaire, the WBQ (Whiplash belief questionnaire) , as previously validated in low back pain. The French WBQ version has already been used to validate the Whiplash Book and later the French version of the same booklet. The WBQ assesses fears and beliefs about the consequences of whiplash, comprising nine items.

b)- Secondary outcomes

Secondarily, the physicians' fears and beliefs were also assessed using a second questionnaire initially validated in non-specific back pain and adapted to neck conditions . The FABQ (Fear Avoidance Belief questionnaire) in its validated French version comprises two independent scales: the physical activity FABQ and work FABQ. The physical FABQ measures fears and beliefs relating to physical activity in general, namely the actions of daily life. It comprises four items with the score varying from 0 to 24. The work FABQ measures fears and beliefs in relation to work activities. It comprises seven items with the score varying from 0 to 42. The term "back pain" was replaced by "neck pain".

Approach to overall management in the emergency department meant the prescriptions and advice delivered to patients who suffer from whiplash injury. It was a secondary endpoint, evaluated in the form of a clinical case.

## 4.3 Design of trial to be conducted

4.3.1 For the physician

***Inclusion visit***

Screening and inclusion will be made by the physician in charge of the study.

The following data will be collected in the case report:

- Demographic data (age, gender,)

- Socio-professional data (place of practice, length and time of practice, medical training or reading)

-Personal or family history of neck pain or Whiplash

- Fear and beliefs questionnaires: WBQ, FABQ.

***Assessments****:*

Once each of the department heads and the different physicians concerned had given their agreement to take part in the study, an initial questionnaire folder (containing the WBQ and FABQ) was sent by email and by post to each of the physicians drawn by randomization. Upon inclusion, demographic data was collected from the physicians (gender, age, place of practice, and length of time in practice), as well as any personal or family history of neck pain or whiplash. The physicians were also asked how often they encountered cases of whiplash.

Information about knowledge of whiplash injury was also sought. This included continuing medical training or recent reading (within the previous 3 years), and in particular knowledge of the different severity grades established by the Quebec Task Force classification , the radiologic recommendations of the Canadian C-Spine Rule , and lastly the latest HAS recommendation on physical therapy in post-whiplash neck pain .

Once the first questionnaire folder had been completed, a second folder was then sent to all physicians participating in the study. The intervention group was instructed to complete the questionnaires at least 48 hours after the intervention, that is, after reading the information booklet. The same items were included as in the initial folder, with an additional questionnaire enabling the intervention group to qualitatively assess the information booklet. If there was no response, reminders were sent by email, then by telephone, and lastly by post.

## 4.3.2 Bias reduction measures

Randomization :

Randomization was performed by computer by drawing lots with random block sizes and was conducted by a statistician working independently of the study. Seven hospitals were drawn to make up the intervention group (reading the booklet), whereas the other hospitals made up the control group (no specific intervention).

# 5- STATISTICS

5.1 Subjects number

To assess the booklet's impact on whiplash beliefs measured by means of WBQ, the sample size estimation was based on comparing the two study arms, with a two-sided Type-I error of 5% and statistical power of 90%. Based on work proposed by Coudeyre *et al.*  and an effect-size defined according to Cohen’s recommendations , 22 subjects would be needed per randomization group in order to highlight an effect-size equaling 1 (which corresponds to a minimal difference of about 5 points for a standard-deviation between 4.78 and 5.37 concerning WBQ) under the previously described assumptions. It was eventually planned to include 24 subjects per randomization group in order to take into account any physician lost to follow up.

5.2 Data analysis

Statistical analyses were performed using Stata software, Version 13 (StataCorp, College Station, TX, US). The tests were two-sided, with α=0.05. Subjects’ characteristics were described for each group as mean±standard-deviation (SD) or median and interquartile range [IQR] for continuous variables, according to statistical distribution, and numbers of physician (%) for categorical variables. To evaluate the impact of a validated information booklet, the primary analysis was performed in line with Vickers and Altman using an ANCOVA with baseline scores as independent variables. Multivariate analyses (random-effects regression models, linear for quantitative outcome and logistic for dichotomous dependent outcome) were carried out after adjusting for parameters fixed according to univariate results and clinical relevance: age, gender, experience, and center (as random effect). For other parameters, randomized groups were compared using the Chi-squared or Fisher's exact test for categorical variables, and Student's t-test or Mann-Whitney test (normality assessed using the Shapiro-Wilk test and homoscedasticity using the Fisher-Snedecor test) for quantitative parameters, as appropriate. These analyses were completed by intra-groups comparisons performed using paired t-test or Wilcoxon test for quantitative parameters, and Stuart-Maxwell test for categorical variables.

**6- STUDY FEASIBILITY AND EXPECTED RESULTS**

6.1 Study feasibility

The number of subjects was set at 24 subjects per group. This recruitment will be among a cohort of 200 physicians.

6.2 Expected results

We expect a significant improvement in physician knowledge and by the way a reduction of their beliefs and an improvement of their satisfaction with the information received.

**7- ETHICS AND LEGAL FACTS**

## 7.1 Usual care

The techniques and methods used in this research are usually conducted, they can be within the scope of research to evaluate routine care as defined by Act No. 2004-806 of 9 August 2004 (Article L1121-1, 2nd paragraph and section R1121-3 the code of public health/ loi n°2004-806 du 9 août 2004 (article L1121-1, 2° alinéa et article R1121-3 du code de la santé publique).

## 7.2 Registration

## Comité de Protection des Personnes

The protocol and the Subject informed form and consent will be submitted to The Independent ethics committee (CPP Sud-Est VI) and written approval from the Chair of the Ethics Committee is required before the initiation of the study.

**CCTIRS et CNIL**

The notification of the approval will be forwarded to the French authority « Traitement de l’Information en matière de Recherche dans le domaine de la Santé » (CCTIRS) and to «  Comission Nationale Informatique et Libertés (CNIL) ». A request for authorization will be sent by the sponsor to CNIL and CCTIRS before the start of the study.

Physicians won’t receive any grants to take part to the study.

The study will be conducted in accordance to good clinical practices and will respect the principles of Helskinki Declaration (Tokyo 2004, revised).

## 7.3 Source documents

Before starting the study, the investigator shall provide the representative of the sponsor of the research a copy of his curriculum vitae dated and signed with registration number of the College of Physicians.

The version of the protocol, annexes will be jointly signed by the investigator and the sponsor representative. If applicable, the scientific manager is also a signatory.

With each new version of the protocol, made necessary by amendments and / or requests of the authorities, a new number and date will be assigned and the same signatures collected.

Each investigator will undertake to fulfill the obligations of the law and conduct research according to Good Clinical Practice.

## 7.4 Quality insurance

The Clinical research assistant commissioned by the sponsor is responsible for inspection of the case report form at regular intervals, according to the monitoring plan of the study, throughout the study to ensure adherence to the protocol, compliance with the source documents, data consistency, and adherence to regulations on the conduct of clinical research.

The Clinical research assistant commissioned must have access to subject’s medical file and other records related to the study required to verify the case report forms of the study.

## 7.5 Quality control

The investigator is responsible for the authenticity of collected data as part of the study and accepts the legal provisions allowing the sponsor of the study to develop a quality control.

The investigator and coordinator agree to make themselves available for the monitoring visits. During this visits, the following documents will be reviewed:

- Informed consent

- Compliance with the protocol and procedures defined therein

- Quality of collected data in the case report forms : accuracy, missing data, data consistency with the source documents

- Product management

## 7.6 Physicians information

Subjects will be informed fully and fairly, in understandable terms, about the objectives, the constraints of the study, the potentials risks involved, and monitoring measures, security, their rights to refuse to participate in the study and the possibility to withdraw at any time. All these information must be listed on the informed consent given to the subjects.

Subjects may at any time exercise the right to access, rectification and opposition to the transmission of electronic data for from the responsible of the study. This right can be exercised directly or through a physician of their choice concerning their medical data (Law of 4 March 2002 on patients' rights and quality of the health system / loi du 4 mars 2002 relative aux droits des malades et à la qualité du système de santé).

## 7.7 CRF report

CRFs are records of data on each subject as defined by the study protocol. Entries on CRFs shall be made complete, legible and correct using a ball-point pen. Any mistakes shall be corrected by drawing a line over the old entry and by initialing and dating next to the correction. The last page of each visit shall be signed and dated by the investigator to indicate the overall responsibility.

## 7.8 Confidentiality

In accordance with GCP and with the national data protection laws, all information concerning the subjects in the study must be treated as strictly confidential by all persons involved in the study including the clinical, medical and statistical monitor.

Data confidentiality is ensured by the use of the first three letters of the name and the first three letters of the first name of the patient on all documents necessary for research associated with a patient number (given in order of inclusion) or deletion by appropriate means. A list of correspondence between the registered members and the patient number will be established and kept by the physician in charge of the study. It will be destroyed at the end of the study.

Physicians will also be informed at their request of the overall research results

## 7.9 Protocol modification

There will be no alterations or changes to the protocol without agreement of all investigators and sponsor.

If such an agreement, the planned changes will constitute an amendment that will be attached to the protocol.

Any amendment must be notified to the ethic committee if the planned changes affect the ethical or medical-scientific study (evaluation criteria, addition of a new center ….). Minor modifications do not require a review of the ethic committee and CCTIRS and CNIL will be informed.

## 7.10 Trial extension

Any extension of the study (deep modification of inclusion criteria, prolongation of treatment and or unexpected therapeutic procedures) will be considered a new trial.

## 7.11 Archiving

The following documents will be archived by the name of the study in the CHU of Clermont-Ferrand to the end of the period of practical use (15 years).

These documents are:

- Protocol and annexes, any amendments,

- Information and consent forms signed (originals)

- Individual data (authenticated copies of raw data)

- Follow-up documents

- Statistical analysis

- Final report of the study

At the end of the period of practical use, all documents to be archived, as defined in the procedure PG.06.005 “Managing the documentation of protocols” of the University Hospital of Clermont-Ferrand will be transferred to central archives and will be under the responsibility of the Hospital for 15 years after the end of the study according to institutional practices.

No destruction can be performed without the consent of the sponsor. At the end of the 15 years, the sponsor will be consulted for destruction. All data, all documents and reports may be subject to audit or inspection.

## 7.12 Final report

The final report of the study will be co-written by the investigator and the biostatistician. This report will be submitted to each co-investigators for advice. Once a consensus has been obtained, the final version must be endorsed by the signature of the investigator and the sponsor addressed as soon as possible after the effective end of the study.

## 7.13 Financing

CHU Clermont-Ferrand.

## 7.14 Publication and data properties

Investigator will have full and unrestricted access to the database with all anonymized data. It is intended to publish the results of the clinical trial collectively (no individual report or publication will be allowed).

Neither the Investigator nor his agents, consultants, associates or employees shall, directly or indirectly, originate, issue or disclose news releases or any type of announcements, whether written or oral, or organize any presentation or issue any publications regarding the study, and/or any information, data, results, inventions or discoveries made or obtained during the study, without the prior written consent of sponsor, which consent shall not be unreasonably withheld.

**8- REFERENCES**

1. Revel M (2003) [Whiplash injury of the neck from concepts to facts]. Ann Readapt Med Phys 46: 158-170.

2. Berecki-Gisolf J, Collie A, McClure R (2013) Reduction in health service use for whiplash injury after motor vehicle accidents in 2000-2009: results from a defined population. J Rehabil Med 45: 1034-1041.

3. Styrke J, Stalnacke BM, Bylund PO, Sojka P, Bjornstig U (2012) A 10-year incidence of acute whiplash injuries after road traffic crashes in a defined population in northern Sweden. Pm R 4: 739-747.

4. Chappuis G, Soltermann B (2008) Number and cost of claims linked to minor cervical trauma in Europe: results from the comparative study by CEA, AREDOC and CEREDOC. Eur Spine J 17: 1350-1357.

5. Jensen TS, Kasch H, Bach FW, Bendix T, Kongsted A (2010) [Definition, classification and epidemiology of whiplash]. Ugeskr Laeger 172: 1812-1814.

6. Buchbinder R, Jolley D, Wyatt M (2001) Population based intervention to change back pain beliefs and disability: three part evaluation. Bmj 322: 1516-1520.

7. Spitzer WO, Skovron ML, Salmi LR, Cassidy JD, Duranceau J, et al. (1976) Scientific monograph of the Quebec Task Force on Whiplash-Associated Disorders: redefining "whiplash" and its management. Spine 20: 1S-73S.

8. Council AGNHaMR (2008) Clinical Guidelines for best practice management of acute and chronic whiplash-associated disorders. Trauma and injury recovery.

9. Buitenhuis J, de Jong PJ (1976) Fear avoidance and illness beliefs in post-traumatic neck pain. Spine 36.

10. Nieto R, Miro J, Huguet A (2009) The fear-avoidance model in whiplash injuries. Eur J Pain 13: 518-523.

11. Coudeyre E, Tubach F, Rannou F, Baron G, Coriat F, et al. (2007) Effect of a simple information booklet on pain persistence after an acute episode of low back pain: a non-randomized trial in a primary care setting. PLoS One 2.

12. Coudeyre E, Demaille-Wlodyka S, Poizat S, Burton K, Hamonet MA, et al. (2007) Could a simple educational intervention modify beliefs about whiplash? A preliminary study among professionals working in a rehabilitation ward. Ann Readapt Med Phys 50: 552-557.

13. Burton AK, Waddell G, Tillotson KM, Summerton N (1976) Information and advice to patients with back pain can have a positive effect. A randomized controlled trial of a novel educational booklet in primary care. Spine 24: 2484-2491.

14. Stiell IG, Clement CM, McKnight RD, Brison R, Schull MJ, et al. (2003) The Canadian C-spine rule versus the NEXUS low-risk criteria in patients with trauma. N Engl J Med 349: 2510-2518.

15. Childs JD, Cleland JA, Elliott JM, Teyhen DS, Wainner RS, et al. (2008) Neck pain: Clinical practice guidelines linked to the International Classification of Functioning, Disability, and Health from the Orthopedic Section of the American Physical Therapy Association. J Orthop Sports Phys Ther 38: A1-A34.

16. ANAES (Novembre 2003) Masso-Kinésithérapie dans les cervicalgies cimmunes et dans le cadre du coup du lapin ou Whiplash. Recommandations pour la pratique clinique.

17. Buchbinder R, Jolley D (2004) Population based intervention to change back pain beliefs: three year follow up population survey. Bmj 328: 321.

18. Gremeaux V, Coudeyre E, Viviez T, Bousquet PJ, Dupeyron A (2014) Do Teaching General Practitioners' Fear-Avoidance Beliefs Influence Their Management of Patients with Low Back Pain? Pain Pract 1: 12248.

19. Darlow B, Fullen BM, Dean S, Hurley DA, Baxter GD, et al. (2012) The association between health care professional attitudes and beliefs and the attitudes and beliefs, clinical management, and outcomes of patients with low back pain: a systematic review. Eur J Pain 16: 3-17.

20. Ferrari R, Russell AS (1976) Survey of general practitioner, family physician, and chiropractor's beliefs regarding the management of acute whiplash patients. Spine 29: 2173-2177.

21. Ng TS, Pedler A, Vicenzino B, Sterling M (2014) Physiotherapists' Beliefs About Whiplash-associated Disorder: A Comparison Between Singapore and Queensland, Australia. Physiother Res Int 23.

22. Rebbeck T, Macedo L, Paul P, Trevena L, Cameron ID (2013) General practitioners' knowledge of whiplash guidelines improved with online education. Aust Health Rev 37: 688-694.

23. Rebbeck T, Macedo LG, Maher CG (2013) Compliance with clinical guidelines for whiplash improved with a targeted implementation strategy: a prospective cohort study. BMC Health Serv Res 13: 1472-6963.

24. Rebbeck T, Maher CG, Refshauge KM (2006) Evaluating two implementation strategies for whiplash guidelines in physiotherapy: a cluster randomised trial. Aust J Physiother 52: 165-174.

25. McClune T, Burton AK, Waddell G (2003) Evaluation of an evidence based patient educational booklet for management of whiplash associated disorders. Emerg Med J 20: 514-517.

26. Waddell G, Newton M, Henderson I, Somerville D, Main CJ (1993) A Fear-Avoidance Beliefs Questionnaire (FABQ) and the role of fear-avoidance beliefs in chronic low back pain and disability. Pain 52: 157-168.

27. Chaory K, Fayad F, Rannou F, Lefevre-Colau MM, Fermanian J, et al. (1976) Validation of the French version of the fear avoidance belief questionnaire. Spine 29: 908-913.

28. Erlbaum L, editor (1988) Statistical power analysis for the behavioral sciences (2nd ed.).

29. Vickers AJ, Altman DG (2001) Statistics notes: Analysing controlled trials with baseline and follow up measurements. Bmj 323: 1123-1124.

30. Coudeyre E, Rannou F, Tubach F, Baron G, Coriat F, et al. (2006) General practitioners' fear-avoidance beliefs influence their management of patients with low back pain. Pain 124: 330-337.

31. Gremeaux V, Coudeyre E, Viviez T, Bousquet PJ, Dupeyron A (2015) Do Teaching General Practitioners' Fear-Avoidance Beliefs Influence Their Management of Patients with Low Back Pain? Pain Pract 15: 730-737.
